# Portable Electronic Nose Based on Electrochemical Sensors for Food Quality Assessment

**DOI:** 10.3390/s17122715

**Published:** 2017-11-24

**Authors:** Wojciech Wojnowski, Tomasz Majchrzak, Tomasz Dymerski, Jacek Gębicki, Jacek Namieśnik

**Affiliations:** 1 Department of Analytical Chemistry, Faculty of Chemistry, Gdańsk University of Technology, 80-233 Gdańsk, Poland; tomasz.majchrzak@pg.edu.pl (T.M.); tomasz.dymerski@pg.gda.pl (T.D.); jacek.namiesnik@pg.edu.pl (J.N.); 2 Department of Chemical and Process Engineering, Faculty of Chemistry, Gdańsk University of Technology, 80-233 Gdańsk, Poland; jacek.gebicki@pg.gda.pl

**Keywords:** agriculture, meat spoilage, vegetable oil, quality assessment, electronic nose, electrochemical sensors

## Abstract

The steady increase in global consumption puts a strain on agriculture and might lead to a decrease in food quality. Currently used techniques of food analysis are often labour-intensive and time-consuming and require extensive sample preparation. For that reason, there is a demand for novel methods that could be used for rapid food quality assessment. A technique based on the use of an array of chemical sensors for holistic analysis of the sample’s headspace is called electronic olfaction. In this article, a prototype of a portable, modular electronic nose intended for food analysis is described. Using the SVM method, it was possible to classify samples of poultry meat based on shelf-life with 100% accuracy, and also samples of rapeseed oil based on the degree of thermal degradation with 100% accuracy. The prototype was also used to detect adulterations of extra virgin olive oil with rapeseed oil with 82% overall accuracy. Due to the modular design, the prototype offers the advantages of solutions targeted for analysis of specific food products, at the same time retaining the flexibility of application. Furthermore, its portability allows the device to be used at different stages of the production and distribution process.

## 1. Introduction

The development and optimization of control methods at each stage of the production process, from raw materials to final products, is becoming increasingly important in food processing technology. There is a growing interest in food safety and food quality assessment issues, which have become one of the main priorities in food analysis. This is evidenced by the novel monitoring and quality control methods both for raw and processed products’ samples.

Apart from classic instrumental techniques (mainly chromatographic), methods based on electronic sensors are increasingly being employed in food analysis [1,2]. In particular, electronic noses are being used for analyses of the samples’ aromatic profiles without prior separation of the volatile fraction into individual components. These devices consist of an array of non- or partially selective gas sensors and are coupled with a data processing and pattern recognition system capable of identifying even complex aromatic profiles [3]. The use of this technique allows rapid non-destructive analysis and in certain applications can be an alternative to relatively costly and time-consuming techniques such as gas chromatography coupled with mass spectrometry (GC-MS) and/or olfactometry (GC-O), infrared spectroscopy (IR), and classical sensory analysis. The last one remains the standard in quality analysis partly because its results can be more directly related to the consumers’ perception of the final product. However, sensory analysis is relatively costly as it requires the participation of trained panellists who can only work for several hours each day due to sensory fatigue [4]. It could be easily supplemented with electronic olfaction, in which also a holistic analysis of the sample’s aromatic profile is performed. Food control is one of the main application areas of electronic noses, in particular in quality assessment and process operations in the food industry [5,6]. In fact, nearly half of the publications in the area of electronic olfaction are related to the food industry [7].

In the past, analytical devices equipped with arrays of sensors, such as electronic noses electronic tongues were developed for analyses of a large variety of food products, e.g., fish [7], meat [8], honey [9], coffee [10], cheese [11], spirits [12], or wine [13,14,15], and also more generic devices for general food analysis [16,17]. The latter ones have been developed into commercially available devices, as less specific solutions can be used in a wider area of applications, despite the fact that a more targeted approach might lead to a higher overall sensitivity and specificity of the system. In this paper, a portable electronic nose intended for food analysis is presented. It is equipped with an array of electrochemical (EC) sensors mounted in easily replaceable modules. In order to adapt it to a particular application, individual sensor modules can be easily changed without the need to modify the platform in any way. Due to its portability, it can be used at all stages of the manufacturing process, whether during monitoring of production, quality evaluation during processing, or at the retail level for freshness assessment. 

## 2. E-Nose System

**Figure 1:**
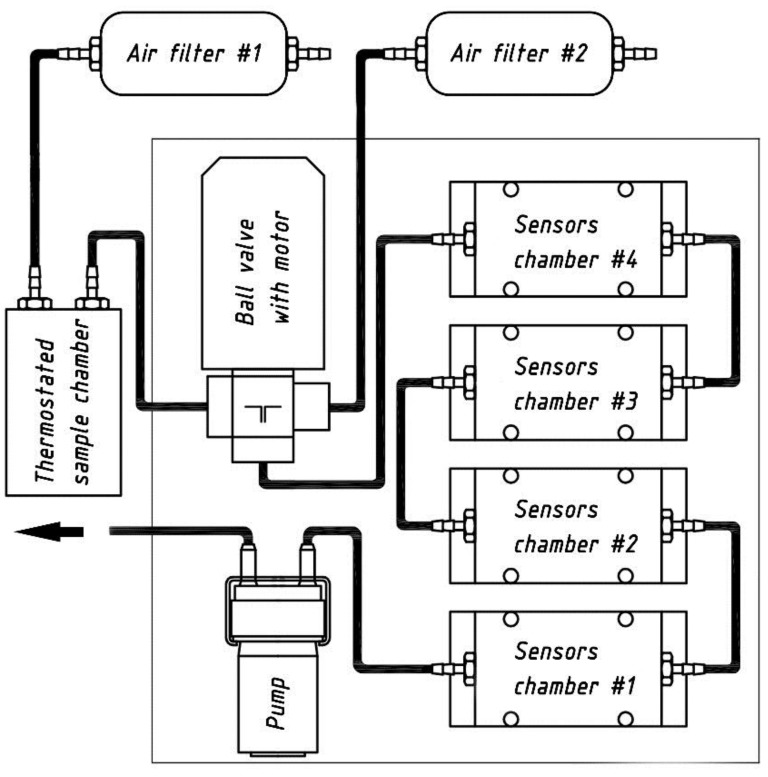
Pneumatic assembly of the electronic nose prototype.

The electronic nose system has been developed to analyse the volatile fraction of various food products. Its pneumatic assembly for dynamic sampling consists of a thermostated sample block, a two-way valve, four replaceable sensor modules connected in sequence, two gas filters, and a pump. A schematic representation of the pneumatic assembly is depicted in Figure 1. 

**Figure 2:**
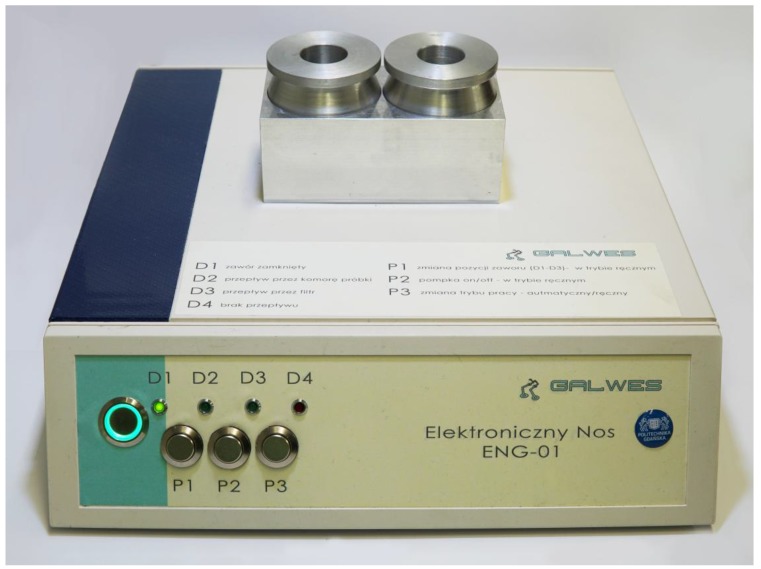
The view of an electronic nose prototype based on the electrochemical sensor array.

The device is controlled through a custom interface printed circuit board (PCB) equipped with a microprocessor, 12 V power supply, signal inputs and outputs, and an analogue-digital signal converter. The main board processes and analyses output signals from chemical gas sensors and other sensors (temperature, relative humidity, pressure) and controls the sample block’s thermostat and pneumatic systems. The processed signals are then transmitted to a personal computer, where the results are displayed in an in-house designed software which can also be used to control the device’s operation. The prototype can also be operated manually using controls and LEDs situated on the front panel of the enclosure (Figure 2).

### 2.1. Sensor Arrays

In food analysis with the use of electronic noses, there is a need to tune the hardware to the specific application [6], which usually means selecting chemical sensors sensitive to the main components of the sample’s volatile fraction. However, regardless of the intended use, there is much overlap in the overall design of the electronic, pneumatic, and data processing systems. For that reason, in order to increase the device’s versatility without losing the advantages of using sensor arrays tailored to the specific application, gas sensors were placed in four easily interchangeable and replaceable modules, with two sensors in each. Moreover, since the circuitry of each module is printed on a custom PCB board, each unit is compact and can be plugged into the main board and removed without much effort. Individual modules are connected in-line, which means that only one needs to be connected for the electronic nose to work. The response delay between the same sensor chamber placed at the beginning and at the end of the series is app. 1 s, which does not affect the outcome of the analysis.

Electrochemical sensors manufactured using screen printing on FR4 plates (SPEC Sensors, Newark, CA, USA) were chosen because of their 10-year estimated lifetime, as opposed to the more usual two-year lifetime. This will improve the system’s robustness and reduce the frequency of maintenance. Two additional electrochemical sensors with an estimated lifetime of two years (City Technology Ltd., Portsmouth, UK) were also used. All the sensors used in the prototype device are listed in Table 1. It can be seen that these electrochemical sensors present cross sensitivity to some chemical compounds. When used as a single sensor for specific compound detection, cross-sensitivity is a real limitation. However, in the case of electronic noses, it increases the range of potential applications of the device. Electrochemical sensors are particularly useful for portable applications due to a low power consumption and the fact that they are relatively unaffected by changes in relative humidity, which is especially important when considering field deployment [18,19].

**Figure 3:**
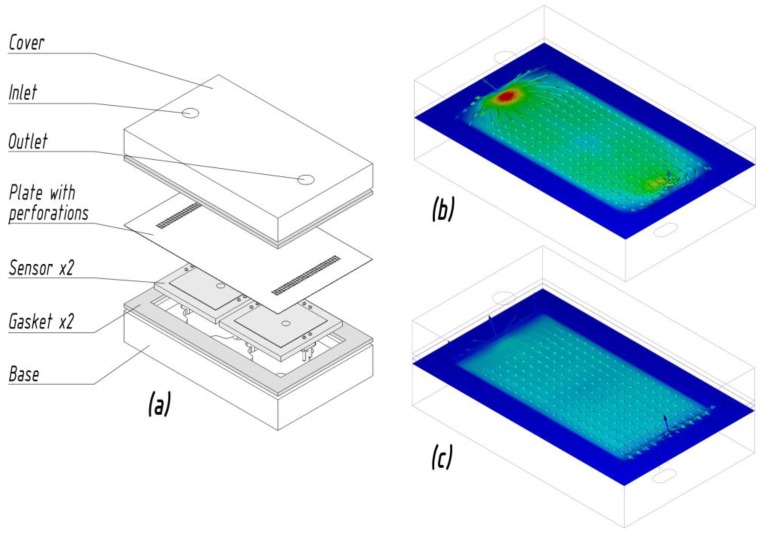
(**a**) Exploded view drawing of the sensor module; (**b**) flow velocity magnitude along the sensors’ surface without the perforated plate and (**c**) with the perforated plate.

In order to improve the response time of the system and facilitate sensor recovery, thus achieving high measurement frequencies, it is important to minimize dead volumes, especially in the sensors chamber [20]. The dead volume in a single module’s sensor chamber is app. 1 ccm. However, reducing the volume above the sensor’s active element might increase the flow turbulence, which in turn might have a detrimental effect on the stability of the response signal. In order to reduce this effect, and even out the flow velocity along the sensors surface, a perforated plate designed using Finite element method Computational fluid dynamics software (Autodesk CFD 2018) was introduced into the sensor modules, as shown in Figure 3.

### 2.2. Sampling System

In order to facilitate rapid consecutive measurements, samples of food products are placed in standard 60 mL or 20 mL glass headspace vials. These can be placed in a thermostated aluminium block operating in the 25 °C to 45 °C range to induce the transfer of analytes to the sample’s volatile fraction. An aluminium adapter is used to accommodate the smaller 20 mL headspace vials. Since the heating block has two slots, an empty vial can be used as a reference. 

There are two basic operating modes, namely ‘sample’, in which the carrier gas (by default ambient air passed through a carbon filter) is directed using a two-way ball valve through the sample’s headspace and into the sensor chamber, and ‘purge’, in which it bypasses the sample and goes directly into the sensor chamber to flush any remaining analytes and condition the sensors before the next measurement. As the sensors’ response characteristic is dependent on temperature and, to a lesser degree, humidity, an integrated sensor which measures these parameters (SHT25, Sensirion, Staefa, Switzerland) is placed in-line. In order to protect the pump in the event of a clog in the pneumatic system, the electronic nose is equipped with an MPX pressure sensor (NXP Semiconductors, Eindhoven, The Netherlands) and the measurement will stop automatically when a threshold pressure level is exceeded.

### 2.3. Data Acquisition System

Signals from the chemical gas sensors and other sensors are processed by the integrated microprocessor, at which point their raw output is converted to a digital signal using the LTC2433 ADC (Linear Technology, Milpitas, CA, USA) and transformed into concentration values (by default ppbv) after temperature corrections are applied. The digital output signal is then transmitted to a PC-class computer via a USB driver (FTDI, Glasgow, UK) and read using custom software written in Python, which enables further processing of the data and also automatic control of the electronic nose’s functions. The user can define the parameters of the method which will be used during the analysis. Apart from recording the sensors’ responses in discrete 1-s intervals, a separate file is created for an entire experiment, consisting of *i* analysed samples, which expedites the subsequent statistical data analysis and pattern recognition. In order to limit the impact of baseline noise on the result of the analysis, the average of n last seconds of the ‘purge’ mode is subtracted from the average of n last seconds of the ‘sample’ mode. All data can be easily exported as comma separated values (csv) to facilitate the system’s integration with the existing data analysis workflows. 

## 3. Materials and Methods 

Two preliminary experiments were conducted to verify whether the prototype e-nose could possibly be used in real-life conditions for animal and vegetable agricultural products analysis, namely meat shelf-life assessment and vegetable oil quality evaluation. 

First, a volatile fraction of fish, pork, and poultry meat that was stored at room temperature over a period of two days was analysed in order to test the behaviour of the sensors and of the data acquisition system. 

In the below-described experiments, a single analysis cycle consisted of 100 s purge time, 20 s sample time and another 100 s purge time and the flow rate was set to 1 dm^3^/min. The measured relative humidity of the air ranged from 50% to 70%.

### 3.1. Poultry Meat Analysis

The prototype was used to determine the shelf-life of refrigerated poultry meat. The fresh chicken breast meat was procured at a local distribution centre in Gdańsk, Poland. The animals were slaughtered in the evening on the day preceding the first day of the analysis, and the carcasses were then transported at 2.4 °C to the distribution centre where they were dismembered. Breast muscles of two different birds were then ground together in a thoroughly cleaned industrial grinder and transported under refrigeration to the laboratory on the morning of the first day of the analysis. Samples of 5 g were placed in 20 mL glass headspace vials, covered with polyethylene foil and stored at 4 °C. Prior to analysis, the vials were sealed with disposable caps lined with a silicon-PTFE membrane. During the analysis cycle, each sample was incubated at 37 °C. 15 samples were analysed every day with a total of 75 samples.

### 3.2. Vegetable Oil Quality Assessment

Refined rapeseed oil, virgin olive oil and unrefined sunflower oil were obtained at a local distribution centre in Gdańsk, Poland. Samples of 5 g were poured into 20 mL glass vials and sealed with a cap lined with a silicon-PTFE membrane. In order to classify samples of rapeseed oil based on the degree of thermal degradation samples were incubated at five different temperatures: 20 °C, 60 °C, 100 °C, 140 °C, and 180 °C, respectively. In total, 50 samples were analysed, 10 for each incubation temperature. 

Subsequently, for virgin olive oil adulteration detection model mixtures of virgin olive oil and unrefined sunflower oil were prepared: 95%, 90%, 80%, and 50% (g/g) of olive oil, respectively. Additionally, samples of 100% olive oil and 100% sunflower oil were also prepared. During the analysis cycle, each sample was incubated at 40 °C.

### 3.3. Statistical Analysis

The chemometric analysis was performed using Orange version 3.6 [21] and Minitab version 17.1 (Minitab, Ltd., Coventry, UK) software. Features were normalised trough centring by mean and scaling by the standard deviation. Features (response signals of particular sensors) most relevant in a particular scenario were then chosen based on the analysis of variance or principal component analysis loadings. Support vector machines method with RBF kernel was chosen as a classifier based on the results of stratified 10-fold cross-validation [22]. Subsequently, the method was evaluated, with 66% of data used for training and 34% for testing through random sampling. 

## 4. Results and Discussion

### 4.1. Meat Shelf Life Evaluation

Presently, commonly used methods of meat freshness assessment are sensory analysis, total volatile base nitrogen (TVB-N) [23] and total bacterial count (TBC) [24]. Each of these methods is time-consuming, requires trained staff, and increases the cost of freshness evaluation. Due to the present regulations and good manufacturing practice, TBC measurement is commonly used in meat production plants. Unfortunately, in this method, a single analysis can take up to 72 h, which means that the batch of meat leaves the production plant before the results of shelf life evaluation are available.

The analysis of volatile compounds, carried out using classical sensory analysis or instrumental methods, can be a source of valuable information regarding meat quality [25,26]. The instrumental analysis used to characterize the aroma of food is usually approached in one of two ways: the first is to examine the individual compounds responsible for the odour of a product, typically by gas chromatography, and the second is based on the so-called holistic aroma analysis. Therefore, it is possible to use electronic noses for this purpose. There is plenty of information in the literature on the use of electronic noses to assess freshness of meat, but there are few solutions that use electrochemical sensors [8]. The following is an example of using this type of device.

**Figure 4:**
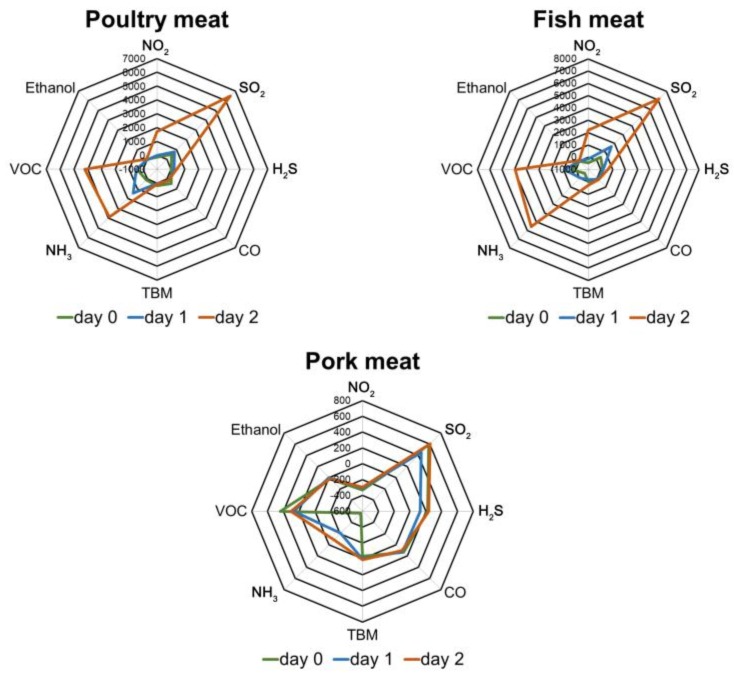
Radar plots for fish, poultry, and pork samples at different time of incubation.

Shelf life differs depending on the type of meat and it is the shortest for fish meat and the longest for pork. Shown in Figure 4 are radar plots of sensor response signals for different types of meat. During the meat spoilage process, the response signals of sensors such as SO_2_, VOCs, and NH_3_ are increased. Poultry and fish significantly deteriorated after two days of storage, whereas pork exhibited similar freshness each day of measurement. In the case of pork, the main constituents of the volatile fraction are compounds that react with SO_2_, H_2_S, and CO sensors. Also, significant negative cross sensitivity signals for the ammonia sensor have been noted for the fresh sample of pork, which also provides valuable analytical information. This preliminary test was intended to check the response signals of the sensors, as simple sensory evaluation is sufficient to assess the freshness of meat stored at room temperature for two days. 

**Figure 5:**
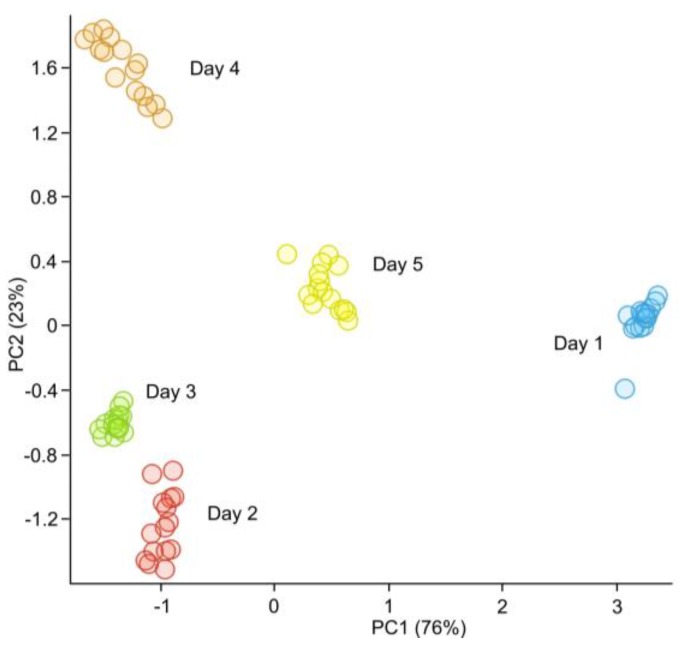
Principal component analysis (PCA) of poultry meat stored at 4 °C over a period of five days.

However, under real conditions fresh meat is stored in temperatures from 2 °C to 7 °C. In these cases, changes in the composition of the volatile fraction with the elapse of storage time is subtle. Therefore, a simple comparison of response signals may not produce satisfactory results and more sophisticated statistical analysis methods are required. In subsequent studies, the possibility to use the prototype electronic nose to classify samples of chicken breast meat based on shelf-life was evaluated. Based on the results of the analysis of variance it was determined, that the signals from four sensors, namely NO_2_, H_2_S, SO_2_, and NH_3_ show the highest correlation with class in this particular scenario. Shown in Figure 5 is the result of principal component analysis of these four features. Two first principal components explain 99% of the total variance.

**Figure 6:**
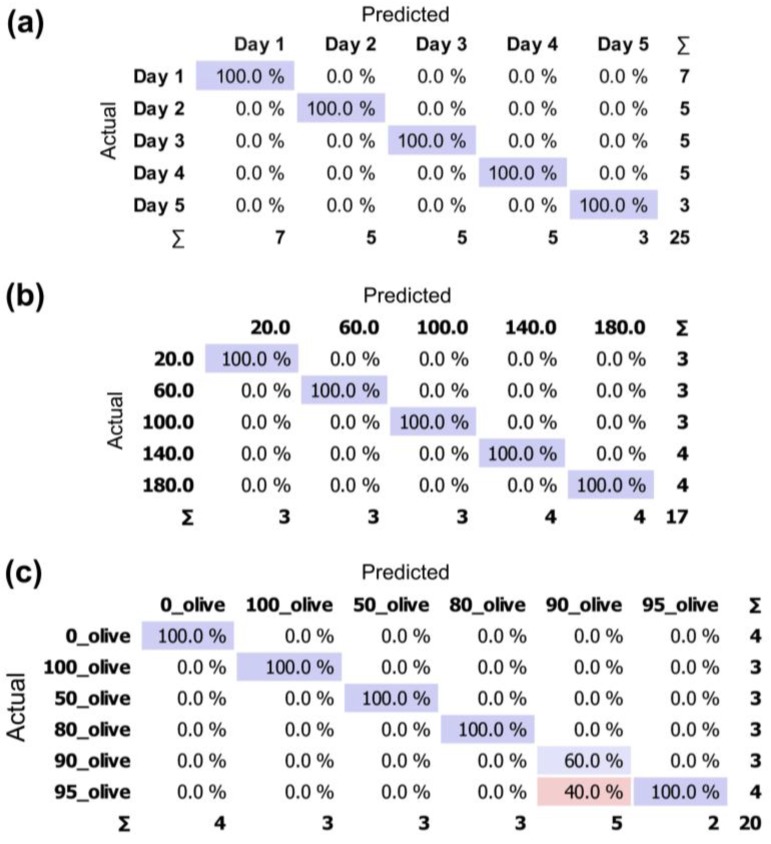
Confusion matrices for Support Vector Machine SVM classification of (**a**) poultry meat based on shelf-life; (**b**) rapeseed oil based on the degree of thermal degradation; (**c**) olive oil based on the admixture of adulterations (purple: classified correctly; pink: misclassified).

Through a stratified 10-fold cross-validation, it was determined that the overall classification accuracy of the SVM algorithm was 98.7%. The SVM method was then trained using 66% of the data chosen at random to avoid bias and tested on the remainder of the data. It was possible to repeatedly and accurately obtain a 100% correct classification based on the shelf-life. A sample confusion matrix for the classification is shown in Figure 6a.

**Figure 7:**
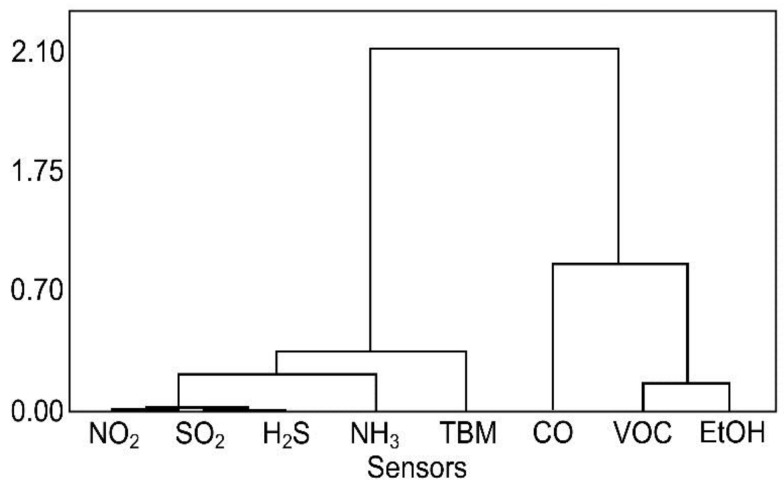
Cluster analysis of variables (sensors) during poultry shelf-life evaluation.

In the same study, cluster analysis was used to determine the groups of sensors that are used in the meat freshness classification. This approach can be used to find correlations between sensor signal values, for example by highlighting a group of compounds that can affect the sensor signal. In order to find the correlation, a cluster analysis using Ward linkage and absolute correlation coefficient distance (Figure 7) was used. It can be noticed that two main clusters are formed. The first one includes sensors that respond to carbon-based substances, namely CO, VOC, and Ethanol sensors. The second cluster contains sensors that detect nitrogen and sulphur compounds, whose presence in the volatile fraction of the meat may induce an unpleasant odour, and which had the greatest impact on the classification result based on ANOVA.

**Figure 8:**
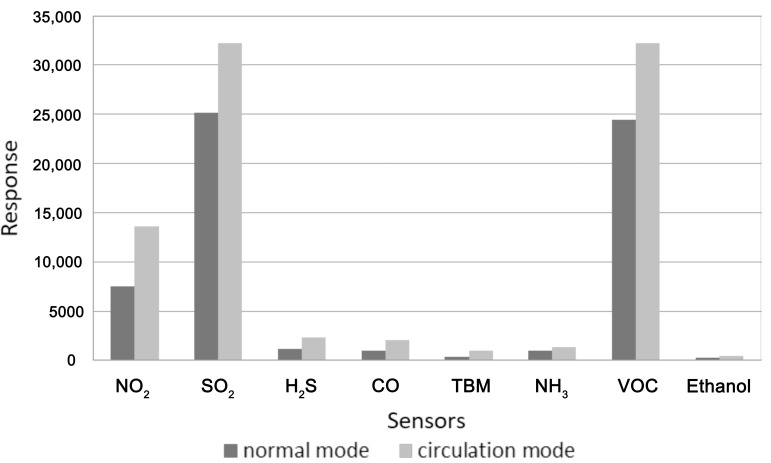
Sensor response signals for poultry meat samples after five days of storage with and without circulation mode.

In subsequent tests, in order to increase the sensor response signal, gaseous sample circulation was used. Unlike the classic DHS, where the carrier gas stream transfers analytes to the detection system, in the proposed approach the flow leaving the sensor chamber is directed back to the sample chamber. As a result, the sensor response signal is increased, as shown in Figure 8. This solution can be found in other agricultural applications, for example in the classification of diluted samples. In the matrices in which volatile chemicals are at high concentrations circulating may cause overloading of the signal and entail measurement errors. It was determined that this procedure does not significantly impact the outcome of classification of meat samples based on shelf-life and the results do not justify increasing the time of a single analysis.

### 4.2. Assessment of the Degree of Vegetable Oil’s Thermal Degradation

The increasing demand for food containing easily digestible micro- and macroelements can lead to a more prevalent use of edible oils for thermal processing of food. Consumption of fried foodstuffs provides assimilable proteins and other nutrients to the human body. Due to the fact that fried foods are characterized by good taste, pleasant aroma, colour, and desirable texture, frying is one of the most popular methods of food preparation [27,28]. Frying occurs when the temperature of oil in which the food is immersed ranges between 150 °C and 190 °C. However, this is a contractual temperature range. In practice, the oil temperature, especially in domestic applications, is not controlled, so the food could be cooked at too low or too high temperatures [29]. This may decrease the quality of the fried product, but it can also be detrimental to consumers. As a result of frying, thermal degradation of oils results in generation of short chain or aromatic volatile organic compounds, e.g., aldehydes such as acrolein, alcohols, ketones, and simple hydrocarbons [30].

Current quality assessment methods, such as sensory analysis, peroxide value measurements, colour determination, and gas chromatography are not in-situ measurement methods and may be labour-intensive and time-consuming. The use of electronic nose devices in which these drawbacks are reduced may be one of the solutions.

**Figure 9:**
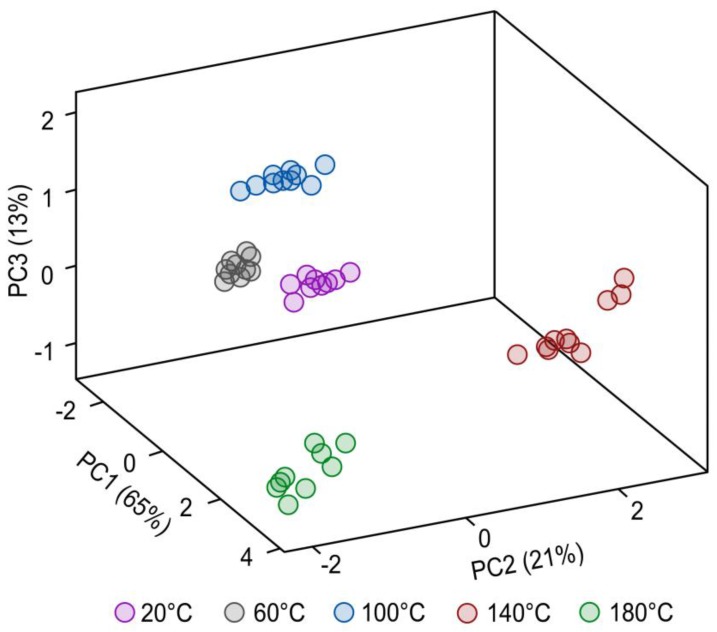
3D projection of PCA of rapeseed oil samples incubated at different temperatures.

Shown in Figure 9 is the result of principal component analysis according to the thermal degradation degree of rapeseed oil samples. The inputs were response signals of all the sensors with which the prototype is equipped. There is a visible separation between samples incubated at different temperatures. Three first principal components explain 99% of the variance. Samples incubated at 20 °C and 60 °C show similarities in the composition of the volatile fraction, as was also demonstrated in previous studies [31]. As the degree of thermal degradation increased, variation within the groups also increased.

**Figure 10:**
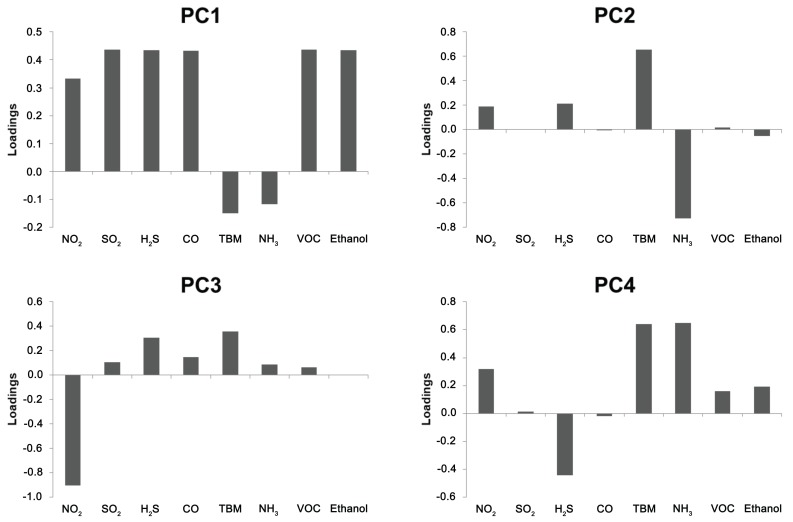
Loadings for the first four principal components in an analysis of rapeseed oil’s thermal degradation.

Analysing the loadings for the principal components, it can be noticed that the first component is determined by an overall increase in the response signal values of all sensors (Figure 10). Classification along the second principal component is based primarily on mercaptan and ammonia sensors’ responses. Thermal degradation products are primarily aldehydes, ketones, and alcohols, so it is to be assumed that the high loading provided by these sensors results more from its cross sensitivity than from the presence of mercaptans and ammonia in the volatile fraction. Similar conclusions can be drawn for other principal components for which the highest loadings are for the nitrogen dioxide sensor for the PC3 and for the mercaptan, ammonia, and hydrogen sulphide sensors for the PC4. In total, the first four major components explain nearly 99.5% of the data variance. Analysis of loadings for major components can provide information on how to limit the number of chemical sensors used. Then, due to the modular nature of the device, it is possible to reduce the number of chemical sensor modules and thereby maintain satisfactory classification results. In the case of the assessment of the degree of thermal degradation, the TBM, NH_3_, NO_2_, and H_2_S sensors had the highest impact on the PCA outcome.

Subsequently, SVM method was used for classification of samples of rapeseed oil according to the degree of thermal degradation. The inputs were features selected based on the interpretation of PCA loadings. As was the case with the classification of meat samples, stratified 10-fold cross-validation was used, and the method was trained using 66% of the data set chosen at random. The use of SVM resulted in 100% correct classification, as shown in Figure 6b. The use of the presented electronic nose to assess the degree of thermal degradation of edible oils allows clear classification. In the future, it is planned to use this device to determine the quality of edible oil during and after the frying process.

### 4.3. Detection of Virgin Olive Oil Adulteration

The choice of vegetable oil for consumption is dictated mostly by its palatability and nutritional properties. Because of concern for consumers’ well-being, the verification of the authenticity of edible oils is one of the key challenges in food analysts [32]. The most commonly adulterated vegetable oil is olive oil. The reason why other oils are added to olive oil is its high price. The inacceptable practice of some oil manufacturers is to admix poor quality oil, which results in an unsatisfactory flavour and nutritional characteristics [33,34]. The most common oil additives are sunflower oil, olive oil of poor quality or hazelnut oil [32,33,35]. The use of electronic noses to detect the adulterations of edible oils, mainly extra virgin olive oil, has been a subject of many research projects [36,37,38,39]. The use of electrochemical sensors in e-nose systems for detecting oil falsification is presented for the first time in this paper.

The SVM algorithm with RBF kernel was used to classify samples of extra virgin olive oil based on the volume of admixed sunflower oil. Based on the results of ANOVA signals from six sensors, namely NO_2_, SO_2_, H_2_S, VOC, CO, and Ethanol were used as features in the statistical analysis. Cross-validation was performed in the same way as in the previously-described experiments, and the classification accuracy was 82.4%. The method was trained on 66% of the data set, chosen at random, and the remaining test set was on average classified correctly with 90% accuracy. A sample confusion matrix for the classification is shown in Figure 6c. It can be noted that misclassifications are contained within the group of samples with 5% and 10% admixture of sunflower oil. It can, however, be determined whether a given olive oil sample was adulterated with 100% accuracy.

## 5. Conclusions

In this article, a portable electronic nose equipped with new-generation electrochemical sensors is described. The use of replaceable sensor modules increases the application potential of this device. Electrochemical sensors are characterized by low electricity consumption, insusceptibility to humidity changes, and long usage life. The use of a perforated plate inside the sensor chamber guarantees the elimination of flow turbulence, which contributes to signal stability, and the easy-to-use interface allows the device to be used even by inexperienced personnel.

Also shown are examples of real-time applications in food quality assessment. The prototype electronic nose was used to assess meat shelf-life, thermal degradation of edible oils, and to detect adulterations of extra virgin olive oil with good results, although the application examples were preliminary in character and will in the future be followed by more in-depth studies of the particular matrixes with tailored arrays of electrochemical sensors. Also, work is underway on developing a hand-held solution in which electrochemical sensors will also be used. Electronic nose systems can in the near future be used to complement the existing food quality assessment methods, in particular in screening tests at the production line.

## Figures and Tables

**Table 1:** Sensors used in the prototype electronic nose.

Sensor	Designation	Manufacturer	Measurement Range	Resolution	Cross-Sensitivity
CO	DGS-CO 968-034	SPEC Sensors	0 to 1000 ppm	100 ppb	Hydrogen, Isopropyl Alcohol
Ethanol	DGS-Ethanol 968-035	SPEC Sensors	0 to 800 ppm	300 ppb	Carbon monoxide, Hydrogen Sulphide, Nitric Oxide, Sulphur Dioxide, Chlorine
H_2_S	DGS-H_2_S 968-036	SPEC Sensors	0 to 10 ppm	10 ppb	Chlorine, Nitrogen Disulphide, Sulphur Dioxide, Nitric Oxide, Carbon Monoxide
NO_2_	DGS-NO_2_ 968-037	SPEC Sensors	0 to 10 ppm	20 ppb	Hydrogen Sulphide, Ozone
SO_2_	DGS-SO_2_ 968-038	SPEC Sensors	0 to 20 ppm	50 ppb	Hydrogen Sulphide, Nitric Oxide, Carbon Monoxide
VOC	DGS-RESPIRR 968-041	SPEC Sensors	0 to 20 ppm	20 ppb	Hydrogen Sulphide, Ozone, Chlorine, Ethanol, Nitrogen Dioxide, Sulphur Dioxide
TBM	2E 50	City Technology	0 to 50 ppm	500 ppb	Nitrogen Dioxide
NH_3_	3E 100 SE	City Technology	0 to 100 ppm	1 ppm	Hydrogen Sulphide
